# Actin bundles play a different role in shaping scales compared to bristles in the mosquito *Aedes aegypti*

**DOI:** 10.1038/s41598-020-71911-0

**Published:** 2020-09-10

**Authors:** Sanja Djokic, Anna Bakhrat, Ido Tsurim, Nadya Urakova, Jason L. Rasgon, Uri Abdu

**Affiliations:** 1 Department of Life Sciences, Ben-Gurion University of the Negev, 84105 Beer Sheva, Israel; 2 Department of Life Sciences, Achva Academic College, Arugot, Israel; 3 Department of Entomology, The Pennsylvania State University, University Park, PA USA; 4 The Huck Institutes of the Life Sciences, The Pennsylvania State University, University Park, PA USA; 5 Center for Infectious Disease Dynamics, The Pennsylvania State University, University Park, PA USA

**Keywords:** Zoology, Entomology, Developmental biology, Body patterning

## Abstract

Insect epithelial cells contain cellular extensions such as bristles, hairs, and scales. These cellular extensions are homologous structures that differ in morphology and function. They contain actin bundles that dictate their cellular morphology. While the organization, function, and identity of the major actin-bundling proteins in bristles and hairs are known, this information on scales is unknown. In this study, we characterized the development of scales and the role of actin bundles in the mosquito, *Aedes aegypti*. We show that scales undergo drastic morphological changes during development, from a cylindrical to flat shape with longer membrane invagination. Scale actin-bundle distribution changes from the symmetrical organization of actin bundles located throughout the bristle membrane to an asymmetrical organization. By chemically inhibiting actin polymerization and by knocking out the *forked* gene in the mosquito (*Ae-Forked*; a known actin-bundling protein) by CRISPR-Cas9 gene editing, we showed that actin bundles are required for shaping bristle, hair, and scale morphology. We demonstrated that actin bundles and *Ae-Forked* are required for bristle elongation, but not for that of scales. In scales, actin bundles are required for width formation. In summary, our results reveal, for the first time, the developmental process of mosquito scale formation and also the role of actin bundles and actin-bundle proteins in scale morphogenesis. Moreover, our results reveal that although scale and bristle are thought to be homologous structures, actin bundles have a differential requirement in shaping mosquito scales compared to bristles.

## Introduction

The vast majority of the epithelial cells that cover insects contain cellular extensions; namely bristles, hairs, and scales. In insects, it has been suggested that thoracic bristles, wing hairs, and scales are homologous structures that differ in their morphology^1,2^. Bristles are elongated and cylindrical in shape, with a different morphology and function than hairs, which are short and shaped like rose thorns, and scales, which are flattened and thin.

Most of our knowledge on the role of actin in bristle^3^ and hair^4^ development are from studies on *Drosophila*. The bristle contains membrane-associated actin filament bundles^5^. It has been shown that two actin cross-linker proteins are involved in bristle actin-bundle formation: the first is Forked, the human espin protein homologue^6,7^; and the second is Singed, the *Drosophila* Fascin homologue, hereafter, referred to in the text as Fascin^6,8,9^. In hair, there are no membrane-associated actin bundles, but instead overlapping cytoplasmic bundles. Organization of actin bundles in the hair is achieved by the sequential use of three actin bundling proteins: Villin, Forked, and Fascin. Thus, bristle and hair generate actin filament bundles, but employ different strategies to assemble these into vastly different shapes^10^. There is little information on the role of actin bundles in shaping insect scales^11,12^. The first paper that described the cytoskeleton organization of scales was on the mill moth (*Ephestia kuenilla*), which revealed two main cytoplasmic elements: actin bundles (referred to in the text as 60 Å fibrils) and microtubules (MTs)^13^. Recently, it was shown that in the butterfly, *Vanessa cardui*, actin bundles in the scales were required for initial scale elongation and to orient the scale parallel to the wing membrane. On the morphological level, actin bundles are required for longitudinal ridge formation and for producing finger-like projections at the tips of wing scales^11^.

Besides *Lepidoptera*, it is also known that, in several mosquito species, the entire body is covered with scales. It was suggested that leg scales may have a role in egg laying by giving the mosquito high water buoyancy and floating ability^14^. In this study, we show that during *Aedes aegypti* pupal development, scales undergo drastic morphological changes. In early pupal stages, the scales are cylindrical in shape, and then they flatten with longer membrane invagination. Actin-bundle distribution changes during development from a symmetrical organization of bundles located throughout the bristle membrane to an asymmetrical organization of round versus flattened bundles. To study the role of actin bundles in scale development, we inhibited actin polymerization during pupal development using chemical inhibitors, and used CRISPR-Cas9 gene editing to knock out the mosquito actin-bundling gene *Forked* (*Ae-Forked*). First, we found that *Ae-Forked* is an essential gene since mosquitoes died as pharate adults (a pharate insect is one that has completed the metamorphosis from larva to adult, but is still within the pupa). We also demonstrated that both in *Ae-Forked* pharate mutant and chemically-treated pupa, scale morphology was altered. We also determined differences in the role of actin bundles in cell elongation of mosquito scales to bristles versus hairs. Our results reveal that the unique organization of scale actin bundles dictate their cellular morphology.

## Materials and methods

### *Aedes aegypti* mosquito rearing

Adult *A. aegypti* mosquitoes were reared in a growth chamber set at 27 °C and 75% humidity on a 12 h light/12 h dark cycle, with unlimited access to water and 10% sucrose solution on a cotton wick. Larvae were reared at 27 °C and 75% humidity in water and fed with a mixture of Tetramin fish flakes and yeast (1:1 ratio). Female mosquitoes were fed on mouse blood using a Hemotek feeder (PS-6 System, Discovery Workshops, Accrington, UK). Blood (3.0 mL) was transferred into the Hemotek blood reservoir unit and the system temperature was set to 37 °C.

### Single guide RNAs (sgRNA) synthesis

The following sgRNA 5′ GGAGTCCACCCTGAAGCCAT 3′ targeting exon 7 in the *Ae-Forked* gene was used. The primers for sgRNA design were annealed using Phusion High-Fidelity DNA Polymerase. sgRNA templates were transcribed using T7 polymerase from a T7 Megascript kit. RNA transcripts were purified using a MEGAclear™ kit.

### Embryo microinjections

*A. aegypti* mosquitoes expressing Cas9 protein under the control of the ubiquitin L40 (AAEL006511) promoter were used^15^. Embryo microinjections were performed as previously described^16^. Briefly, four days after blood feeding, ten mated gravid females were transferred into plastic tubes with oviposition substrate, and kept in the dark for oviposition. After 30 min, eggs were collected and aligned on a piece of Whatman filter paper. Eggs were covered with a 1:1 mix of Halocarbon 700 oil:Halocarbon 27 oil to prevent desiccation. Quartz needles were pulled using a Sutter P2000 needle puller and were used with a Femtojet injector (Eppendorf) and InjectMan micromanipulator. 0.2 nL sgRNA at 400 ng/µL was injected into the embryos. After injection, embryos were allowed to recover under insectary conditions for 5 days before hatching them under a vacuum into water.

### Molecular analysis of mutant individuals

G_0_ adults were visually screened for bristle and scale defects. Individual legs from putative mosaic and non-mosaic edited G_0_ mutants or G_1_ and G_2_ mosquitoes were collected and genomic DNA was extracted using Wizard Genomic DNA Purification Kits or Qiagen DNeasy Blood and Tissue kits. Genomic DNA was used as a template for PCR with the following primers: Forward 5′ CTGTGGGACCCCCACCGCCA 3′ and reverse 5′ CTGATATAATGGACATGCTT 3′. PCR products were separated in 3% agarose gel electrophoresis, eluted from an excised band of the gel, and directly sequenced.

### Pupa phalloidin and antibody staining

For examination of bristles and scales, 0–1-h old pupae were collected from controls (untreated pupae), cytochalasin D-treated pupae, or *Ae-Forked* pupae (from a cross between heterozygous *Ae-Forked* mosquitoes) and reared individually. At the appropriate developmental time after pupal formation (as mentioned in the text), pupal cuticles were removed, and the pupae were fixed for confocal or electron microscopy. In the case of *Ae-Forked* pupae, part of the abdomen was taken for DNA extraction and analyzed as described in molecular analysis of mutant individuals. For confocal microscopy, pupae were fixed in 4% paraformaldehyde in PBS overnight. The samples were washed three times with 0.3% Triton X-100 in PBS for 10 min each time. For phalloidin staining, samples were washed three times with 0.3% Triton X-100 in PBS for 10 min each time, and then incubated overnight with phalloidin. Then the samples were washed three times in 0.3% Triton X-100 in PBS. For antibody staining, the thoraces were blocked in 0.1% Triton X-100 containing 4% bovine serum albumin for 1 h. The samples were then incubated overnight with a primary antibody in the blocking solution at 4 °C, washed three times in 0.3% Triton X-100 in PBS, and incubated with secondary antibodies in blocking solution for 2 h at room temperature or at 4 °C overnight in the dark. After incubation, samples were washed three times with 0.3% Triton X-100 in PBS for 10 min each time. For confocal observation, both phalloidin and antibody-stained samples were placed on a slide and mounted in 50% glycerol. A coverslip was placed on the sample, and the preparation was sealed with nail polish. The slides were examined with an Olympus FV1000 laser-scanning confocal microscope. Mouse anti-α-tubulin (1:250) (Sigma) primary antibodies were used. Goat anti-mouse Cy2 and Cy3 and goat anti-rabbit Cy3 (Jackson ImmunoResearch) secondary antibodies were used at a dilution of 1:100. The goat anti-rabbit Cy3 (Molecular Probes) secondary antibodies were used at a dilution of 1:500. For actin staining, we used Oregon Green 488- or Alexa Fluor 568-conjugated phalloidin (1:250) (Molecular Probes).

### Injection of inhibitors

Stock solution of 5 mg/mL cytochalasin D in DMSO was prepared. Twenty *A. aegypti* pupae at the appropriate developmental time after pupal formation (as mentioned in the text), were injected with 2 nL of cytochalasin-D into the first or second abdominal segment. Control pupae were injected with DMSO. Pupae were allowed to develop and then were prepared for SEM analysis. Each experiment was repeated three times.

### Scanning electron microscopy (SEM)

Samples were fixed and dehydrated by immersion in increasing concentrations of ethanol (30%, 50%, 75%, and twice in 100%; 10 min each). The samples were then completely dehydrated using increasing concentrations of hexamethyldisilazane (HMDS) in ethanol (50%, 75%, and twice in 100%; 2 h each). The samples were air dried overnight, placed on stubs, and coated with gold. The specimens were examined with a scanning electron microscope (SEM; JEOL model JSM-5610LV). Length measurements of adult bristles were performed using Image J (https://rsb.info.nih.gov/ij/) (version 1.40j) software.

### Bristle and scale measurements and statistics

Bristles and scales were examined by scanning electron microscope (SEM). The length and width of bristles and scales were measured from SEM images by using Image J. The length of the bristles were measured from the base of the bristle (only in cases where we could detect the socket cells on the epidermis) up to the bristle tip. In both cytochalasin-D-treated and *Ae-Forked *mutant mosquitoes with split bristles, the measurement was restricted to the main bristle shaft only. The length of the scale was measured from the attenuation at the base till the distal part. The width of the scale was measured both at the middle part and also at the tip. For each individual, we measured 3–6 different scales or bristles (see tables for the exact number) and used the average measurement for the statistical analysis. The difference between wild-type and treated individuals (either cytochalasin-D or *Ae-Forked* CRISPR mutants) was than analyzed using *t* tests with unequal variance estimates. For TEM actin-bundle measurements, the number of actin bundles in each sectioned scale photograph was counted. For the actin-bundle area, in each section, a line that crossed the middle of the scale section was drawn, and the area of each actin bundle was measured by Image J. The difference between each actin-bundle area size group from TEM analysis was then analyzed using *t* tests for two independent samples, assuming unequal variances. The width of scales from confocal microscope analysis was measured by an Olympus FV-1000 program, and the difference between wild-type and treated individuals (either cytochalasin-D or *Ae-Forked* CRISPR mutants) was then analyzed using an ANOVA followed by a Bonferroni post-hoc test.

### Transmission electron microscope (TEM)

Legs from each developmental stage pupa were dissected as described above and fixed for 20 min in 2% glutaraldehyde in 0.2 M PO_4_ (pH 6.8) at room temperature and then for 1 h on ice. After 1 h, the specimens were transferred to a fixative comprising cold water, 0.2 M PO_4_ (pH 6.2), 4% osmium tetroxide (OsO_4_), and 1% glutaraldehyde and placed on ice for 1 h. The specimens were then washed three times in cold water (20 min each) and incubated in 1% uranyl acetate overnight at 4 °C. This was followed by transfer to a dehydration series spanning from 30 to 100% acetone, with 10% increases being made at each 15-min interval. Samples were then treated twice in propylene oxide for 15 min and soaked for 1 h in a 1:1 solution of propylene oxide and araldite, followed by overnight incubation at 4 °C in a 1:2 propylene oxide/araldite mixture. The tissues were then transferred to araldite and incubated for 1 h, placed on araldite blocks (the blocks were polymerized the previous day at 60 °C), and embedded in araldite. These were then left at room temperature for 30 min after embedding, at which point the samples were oriented and incubated at 60 °C for 24 h. Transverse sections of 70 nm were cut through the thorax using a Leica UltraCut UCT ultra microtome equipped with a diamond knife, stained with uranyl acetate and lead citrate, and then examined with a JEOL JEM-1230 transmission electron microscope operating at 120 kV.

## Results

**Figure 1:**
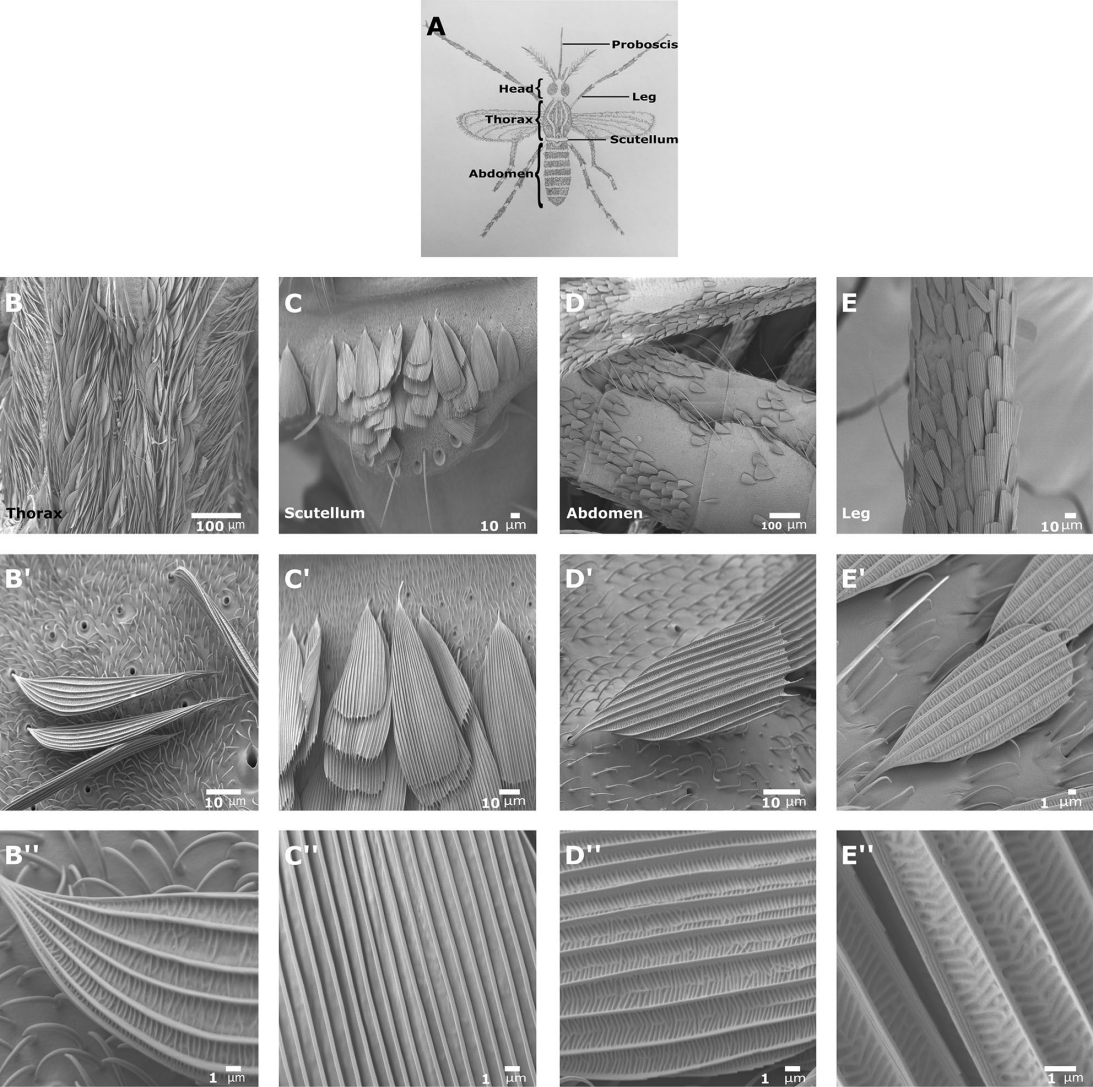
Scanning electron microscopy (SEM) images of scale from different tissues of the mosquito, *Aedes aegypti*. (**A**) Schematic dorsal view of the general external features of an adult mosquito. SEM images of mosquito body parts; (**B**) thorax, (**C**) scutellum, (**D**) abdomen, (**E**) leg. Closer examination of the scales reveals that scales on the thorax (**B**′) are a falcate type, and on the scutellum (**C**′), abdomen (**D**) and leg (**E**′) are a spatulate type. Along each scale, there are longitudinal ridges which spread from the base of the scale to the tip (**B**″,**D**″,**E**″). Except for scales on the scutellum (**C**″), in all other scales between the longitudinal ridges, there are many cross ribs. Scale bars are found on each image.

To understand the role of actin on mosquito scale development, we focused our analysis on four different body sections (Fig. 1A), namely, the thorax (Fig. 1B,B″), scutellum (Fig. 1C,C″), abdomen (Fig. 1D,D″), and legs (Fig. 1E,E″). The mosquito thorax is covered with falcate scales (Fig. 1B), which are curved with a sharp or narrowly rounded apex (Fig. 1B′). In the scutellum, we found that each of the three scutellar lobes (Fig. 1C) contained clusters of five bristles each with its associated spatulate scales, which are lamellar characterized by a broad distal section and attenuation at the base (Fig. 1C′). Also, the abdomen (Fig. 1D) and the legs (Fig. 1E) are covered with spatulate scales (Fig. 1D′,E′). As previously described^14^, along each scale, there are longitudinal ridges spreading from the scale base to tip (Fig. 1B′,C′,D′,E′). Closer examination revealed that only the thorax (Fig. 1B″), abdomen (Fig. 1D″), and legs (Fig. 1E″), but not the scutellum (Fig. 1C″) scales had cross ribs between the ridges.

**Figure 2:**
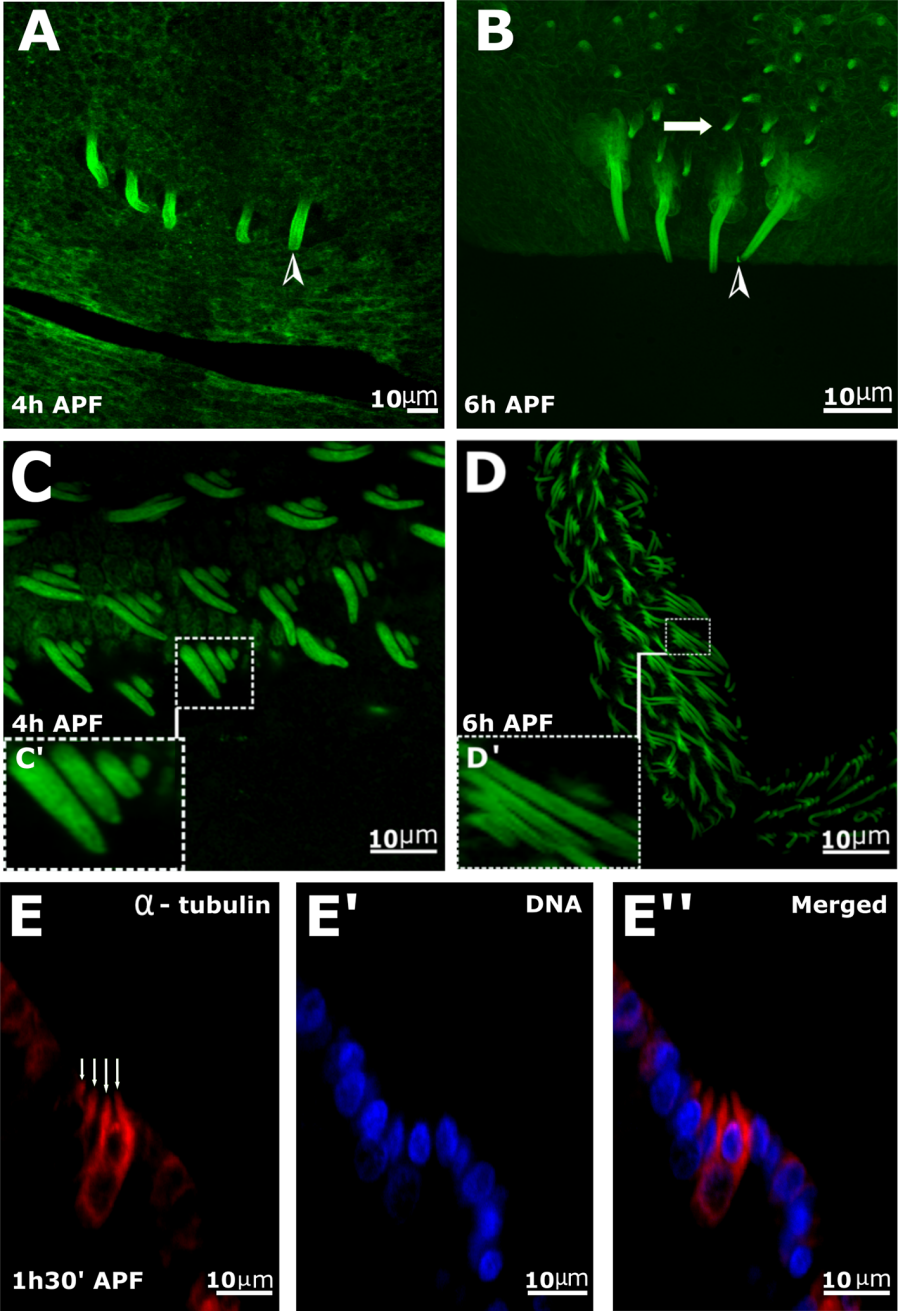
Confocal images of mosquito scale development. (**A**–**E**) Anti-tubulin staining [Tubulin—green (**A**–**D**), and red in (**E**), DNA—blue in **E**′] of scutellum (**A**,**B**) and leg (**C**–**E**) from mosquito pupae at different developmental times [(**A**,**C**) 4 h after pupal formation (APF), (**C**,**D**) 6 h APF, (**E**,**E**″) 1.5 h APF). At 4 h APF, small buds of bristles, but not scales, can be detected (**A**, arrowhead) and at 6 h (**B**), both bristles (arrowhead) and scales (arrow) could be detected on the scutellum. Leg of pupa 4 h (**C**), and 6 h APF (**D**), scales in clusters of four can be seen (**C**′,**D**′). (**E**) At 1 h 30 min. APF, in all four scale cells within one cluster (arrows in **E**), growing out could be detected.

Next, using MT staining, we followed both legs and scutellum scale bud initiation (Fig. 2). We found that on the scutellum, bristles emerged as early as 4 h after pupa formation (APF) (Fig. 2A, arrowhead), but buds of scales were detected only at 6 h APF (Fig. 2B, arrows). On the legs, scales arose as little buds as early as 4 h (Fig. 2C) APF. Scales on the legs were found in clusters of four (Fig. 2D), and in each cluster, the scales differed in size (Fig. 2C′,D′). We noticed that each single cluster was aligned according to size, where the axis of this alignment appeared to point in the same direction, indicating the planar polarity of the cells. Our results demonstrate a difference in timing of scale development between body sections, as well as a difference in scale and bristle development in the same section.

We were intrigued by the organization of the scales on the legs as a cluster of four cells, and decided to investigate their initial growth timing. At 1 h APF (data not shown), we were unable to identify the putative scale cells, but at 1 h 30 min APF, we could detect scale cells growing out from all four cells (Fig. 2, arrows in E).

### Ultrastructure analysis of the mosquito scale

**Figure 3:**
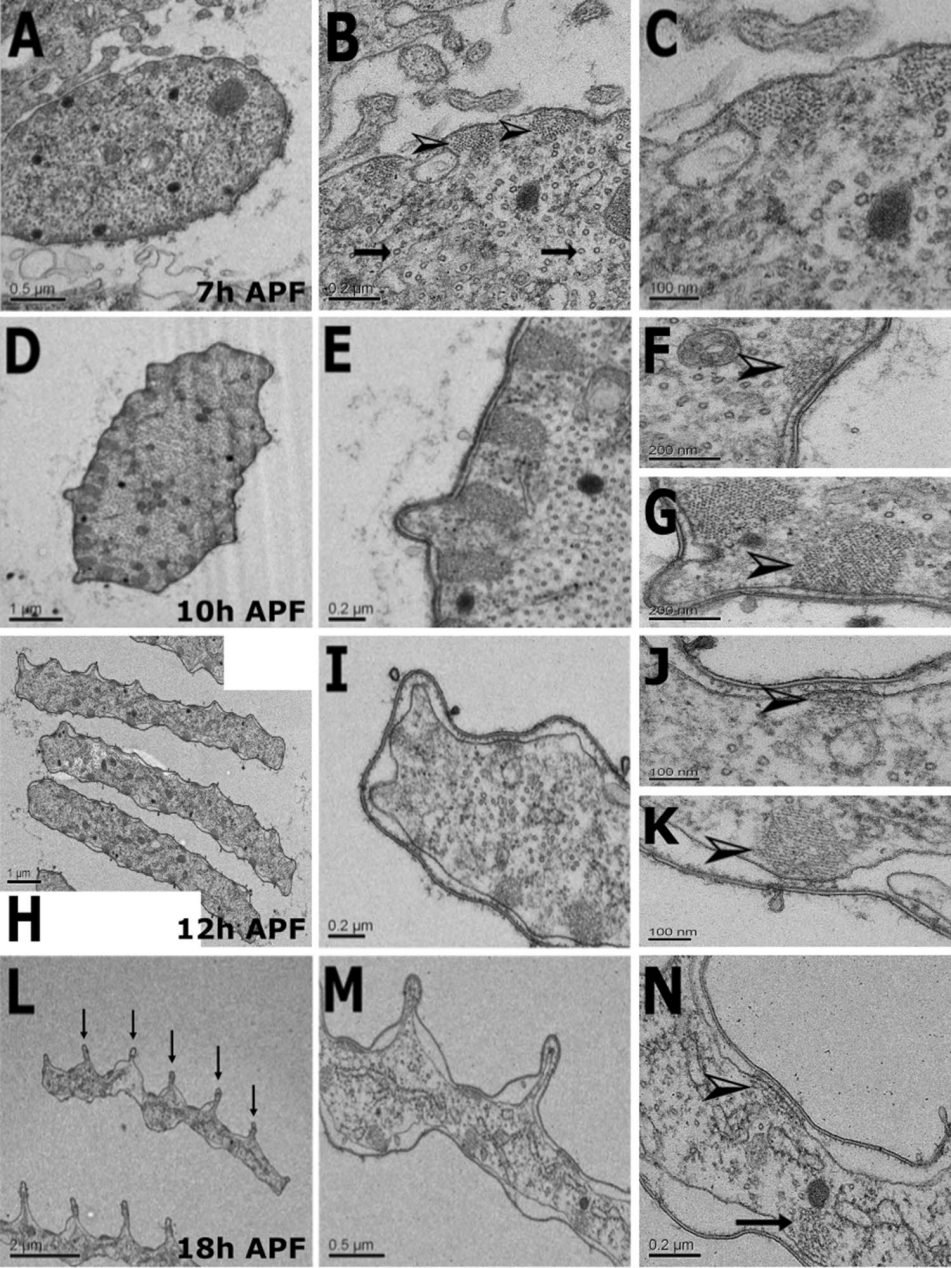
Transverse transmission electron microscopy section of *Aedes aegypti* scales from the leg during development. (**A**–**C**) Scales at 7 h APF show (**B**) centrally positioned microtubules (arrows) and actin bundles (arrowheads) attached to a plasma membrane. (**D**–**G**) At 10 h APF, actin bundles are organized asymmetrically. (**H**–**K**) 12 h APF scales are flattened and elongated, and actin bundles are organized in an asymmetrical manner (**J**). Actin bundles are smaller on one side of the scale (arrowhead) (**K**), and much larger on the other side of the same scale (arrowhead). (**L**–**N**) In scales at 18 h APF, one side of the flattened scales contains obvious ridges and valleys (arrows). These are on the side with flattened actin bundles. (**N**) At this stage, actin bundles have the same pattern as the smallest actin bundles (arrowhead) and largest actin bundles on the other side (arrows). Scale bars are found on each image.

To follow ultrastructure changes of leg scale development, we used TEM analysis. We chose to use legs for EM analysis since this body part is easier to handle compared to the thorax. At 7 APF (Fig. 3A–C), scales were cylindrical in shape, resembling bristles, with membrane-associated actin bundles (Fig. 3B, arrowheads) and dense MTs were found distributed over the scale cytoplasm (Fig. 3B, arrows). The scale contained an average of 19 ± 1.55 (n = 3 pupae, three scales from each pupa), rounded actin bundles that were similar in size throughout the scale membrane (Fig. 3A–C). At 10 h APF (Fig. 3D–G), scales were still rounded with a dense MT network all over, but this time, the bundles were organized asymmetrically with one side of the scale containing large triangular bundles (Fig. 3E, arrowhead), with much smaller rounded ones on the opposite side (Fig. 3F, arrowhead). To quantify this symmetrical actin-bundle organization, we drew a line through the middle of each section through the large diameter of the scale and measured the area of each group of actin bundles from three different pupae and one scale from each pupa. This analysis was done for all time points. We found that, within each scale at 10 h APF, a highly significant difference (*t* test for two-independent samples; p < 0.0001) between actin-bundle areas was found. The average area of the largest actin bundles was 0.06 ± 0.01 µm^2^ as compared to the smallest one, 0.02 ± 0.01 µm^2^. At 12 h APF (Fig. 3H–K), the scales became flattened. The highly significant (p << 0.0001) asymmetrical organization of the actin bundles was still evident, where the average area of the largest actin bundles on one side was 0.04 ± 0.001 µm^2^, and the smallest was 0.01 ± 0.001 µm^2^. At 18 h APF (Fig. 3L–N), the flattened scales had larger ridges (Fig. 3L, arrows) on one side. Again, the highly significant (p << 0.0001) asymmetrical organization in the actin-bundle area was evident (large—0.03 ± 0.001, small—0.01 ± 0.002).

### Inhibition of actin polymerization by cytochalasin-D injection affects bristle and scale development

**Figure 4:**
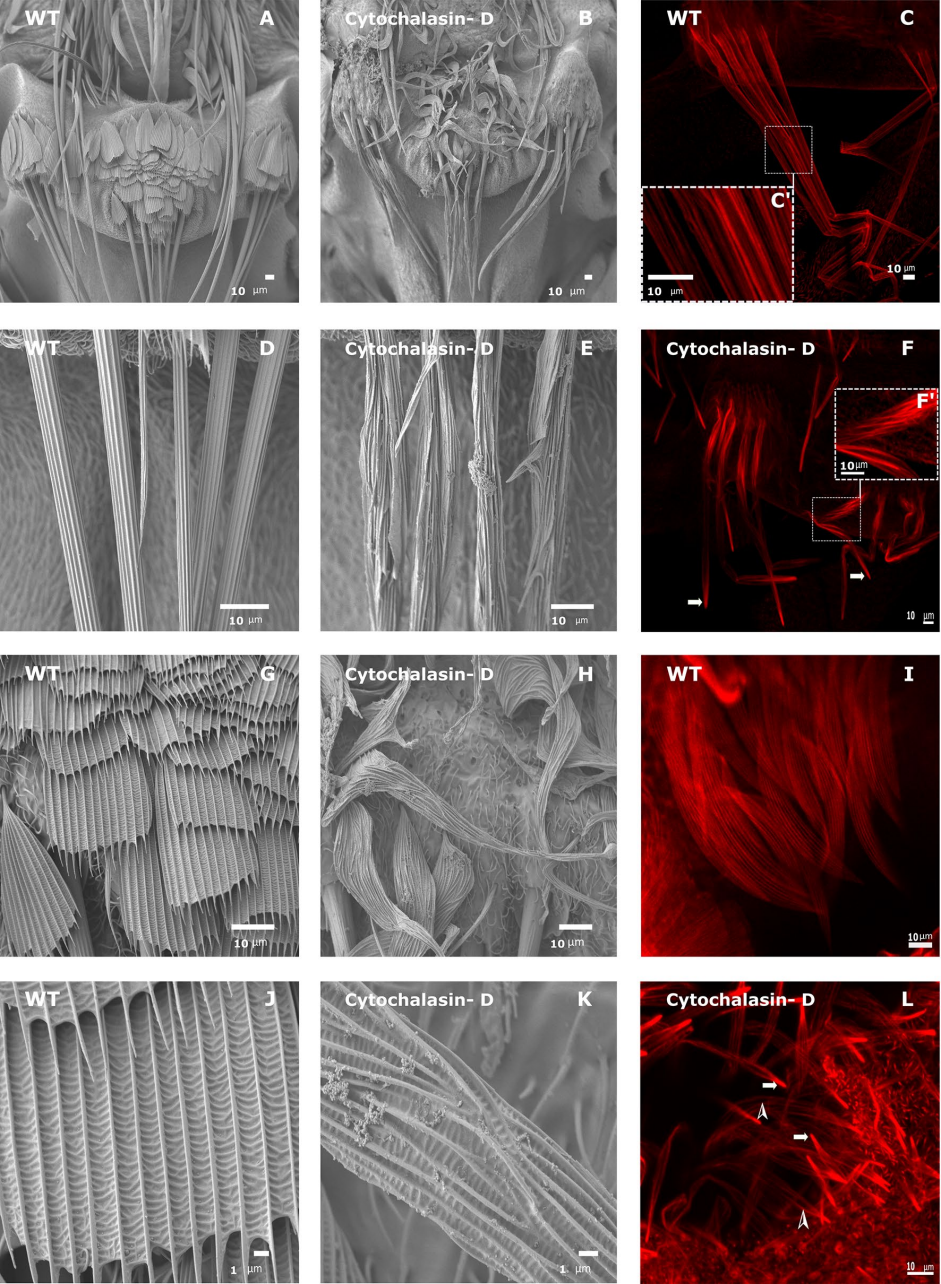
Scanning electron microscopy (SEM) and confocal microscopy images of wild-type (WT) and cytochalasin-D (CD) treated mosquito *Aedes aegypti*. SEM image of scutellum region from WT (**A**) and CD-treated (**B**) mosquitoes. Overall, the bristles are much shorter, and the scales are twisted in CD-treated mosquitoes. Closer examination of bristles from CD-treated mosquitoes (**E**) revealed misorientaion of the ridge pattern compared to WT (**D**). (**C**,**F**) Phalloidin staining (actin-red) of bristle from the scutellum region of (**C**) WT and (**F**) CD-treated *Ae-Forked* pupa 15 h APF. In untreated mosquitoes (**C**,**C**′), actin bundles run parallel to the bristle shaft, where in CD-treated *Ae-Forked* pupae (**F**,**F**′), actin bundles are misoriented (**F**′), and actin accumulates at the bristle tip (**F**, arrows). SEM analysis of scales from CD-treated mosquitoes (**H**) revealed that the WT-like spatulate shape (**G**) is lost in CD-treated mosquitoes and now is twisted and narrower, and the upper part was no longer broad, but instead tapered towards the distal part of the scales. Also, misorientation of the ridge pattern in CD-treated (**K**) mosquitoes compared to WT (**J**) is detected. Scale bars are found on each image. Phalloidin staining (actin-red) of scales from the thorax region of (**I**) WT and (**L**) CD-treated *Ae-Forked* pupae 15 h APF revealed an abnormal accumulation of actin at the scale tip (arrows), and that actin bundles were no longer evenly distributed, and the shaft contained regions lacking actin bundles (arrowhead).

To study the role of actin in bristle and scale development, cytochalasin D (CD) (which inhibits actin polymerization by capping the barbed end of actin filaments and inhibits further elongation from the barbed end^17^ was injected into the pupae. Previously, it was shown, that injection of CD into pupae affects *Drosophila* bristle growth^18,19^. We focused analysis on the scutellum bristles and scales. Since we found that, on the scutellum, initiation of bristles and scales starts after 4 h and 6 h, respectively (Fig. 2), we injected CD into 4 h AFP pupae. We found that the bristle length (148.791 ± 8.803 µm) of treated pupae (Fig. 4B) was significantly shorter (t = − 7.101, df = 6, p = 0.0004) than in wild-type bristles (Fig. 4A; 255.082 ± 12.106 µm; Table 1). The overall morphology of the bristles were affected—instead of a long tapered cylinder shape with a regular ridge and valley pattern (Fig. 4D), the bristles were blunt with splits along their length (Fig. 4E), and the ridge and valley pattern was misoriented (Fig. 4E). Next, we examined the effect of CD on bristle and actin-bundle organization during pupa development by confocal microscope analysis following phalloidin staining. We stained 15 h APF pupae, when both bristles and scales are fully elongated, and found that, in bristles on the scutellum of untreated pupae, parallel actin bundles ran along the entire bristle shaft (Fig. 4C,C′). On the other hand, in CD-treated pupae, the actin bundles misoriented throughout the bristle shaft (Fig. 4F,F′), with strong accumulation of actin at the bristle tip (Fig. 4F, arrows).

**Table 1:** Bristle and scale measurement in WT and cytochalasin-D-treated pupae.

Genotype	WT	Cytochalasin-D
No. of pupa	4	9
No. of bristles	15	30
Bristle length	255.082 ± 12.106*	148.791 ± 8.803
No. of pupa	6	9
No. of scales	21	34
Scale width- middle	25.385 ± 1.204*	7.029 ± 1.072
Scale width-top	23.718 ± 0.960*	1.09 ± 0.111
Scale length	56.789 ± 3.891*	77.27 ± 5.195

*Represents a significant difference (p < 0.05) between genotypes (for a detailed description of statistical analysis performed, see “Materials and methods”).

Then we examined scale morphology, and found clear defects in the morphology. Overall, the scales were curved in shape (Fig. 4H), and their length was significantly (t = 3.156, df = 13, p << 0.0076) longer (77.27 ± 5.195 µm) than the wild type (56.789 ± 3.891 µm; Table 1). Moreover, the scales had lost their spatulate shape (Fig. 4G vs. Fig. 4H), where the upper part was no longer broad, but instead tapered towards the tip, resembling the bristle tip. They were significantly narrower (t = − 11.385, df = 12, p << 0.0001) than the wild type, both at the middle; (7.029 ± 1.072 µm vs. wild type = 25.385 ± 1.204 µm and t = − 23.406, df = 5, p << 0.0001 at the tip; 1.09 ± 0.111 µm as compared to wild type = 23.718 ± 0.960 µm; Table 1). Closer examination of the scales revealed that the longitudinal ridges (Fig. 4J) were misoriented in the CD-treated mosquito (Fig. 4K). Since our attempt to stain actin bundles in scales from the scutellum was unsuccessful, we focused our analysis on scales on the thorax. We showed that in untreated pupae, equalized size and evenly distributed actin bundles ran in parallel throughout the scale length (Fig. 4I). However, in CD-treated pupae (Fig. 4L), the scales were significantly narrower (p < 0.0001, WT—7.6 ± 1 µm n = 15 scales from four pupae; CD-treated—5.4 ± 0.7 µm, n = 12 scales from four pupa) than untreated scales (Fig. 4K). Moreover, higher accumulation of actin staining was found on the upper part (Fig. 4L, arrows) and on the lower part of the scales, actin bundles were no longer evenly distributed, and the shaft contained regions lacking actin bundles (Fig. 4L, arrowheads).

### Forked is required for both mosquito bristle and scale development

**Figure 5:**
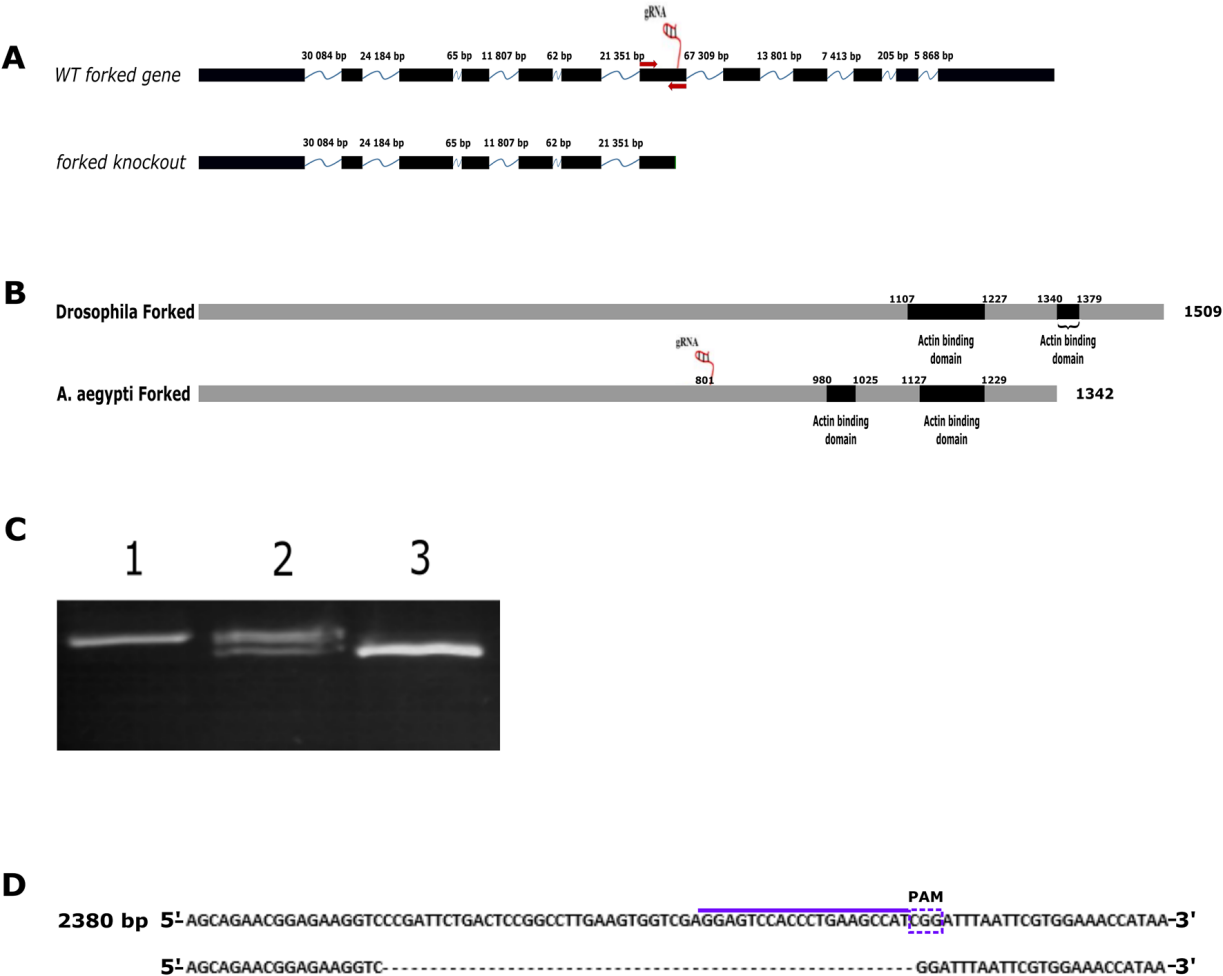
Generation of *Ae-Forked* mutant using CRISPR/CAS9 system. (**A**) Genomic organization of the *Ae-Forked* gene. The black boxes are exons, the blue line represents introns, and the number above them is the intron size in base pairs. sgRNA on exon 7 is marked with a box; primers that were used to characterize (PCR results in **C**) *Ae-Forked *mutants are marked in red. sgRNA was injected into *A. aegypti* transgenic mosquito embryo ubiquity expressing CAS9 protein under *ubiquitin L40 *(AAEL006511) promoter were used^15^. (**B**) Drosophila and *A. aegypti* *Forked* protein. The *Drosophila Forked* protein contained two actin-binding sites (black boxes), 1,107 to 1,227 aa and 1,340 to 1,379 aa^20^. These actin-binding domains (black boxes) are located from amino acid 980 to 1,025 and from amino acid 1,127 to 1,229 aa in *Ae-Forked* protein**.** Our truncated *Ae-Forked* mutant protein, which contains only 801 aa, lacks these domains. (**C**) PCR analysis of WT and mutant mosquito lines. (1) PCR on WT mosquitoes reveals PCR product of 414 nt. (2) PCR on G1 putative mutant heterozygous lines reveals two PCR products, one the same size as in WT, and the second smaller in size, about 350 nt. (3) PCR on homozygous G2 *Ae-Forked* line showing only one band which represents the deletion of 52 bp. (**D**) DNA sequence of genomic DNA from WT *Ae-Forked* around the sgRNA site (the upper line at the purple end of the PAM site is marked with a box). Below is the DNA sequence of genomic DNA from the *Ae-Forked* mutant line showing deletion of 52 bp. The full blot summary can be found in the Supplementary Information.

To further investigate the role of actin bundles in scale development, we mutated one of the known *Drosophila* bristle and hair actin bundles genes—*Forked*. Blast analysis revealed the annotated gene AAEL018112 (hereafter, *Ae-Forked*) was homologous to the *Drosophila Forked* gene. To generate the *Ae-Forked* mutant line, a sgRNA targeting exon 7 (Fig. 5A) was injected into eggs of a transgenic mosquito line that expressed Cas9 under control of the ubiquitin L40 promoter^15^. Injection of sgRNA into 200 embryos yielded 26 Generation 0 (G0) mosquitoes. All of the G0 mosquitoes were crossed to wild-type mosquitoes of the opposite sex. Next, eggs from one of the G1 generation lines were hatched, and all 50 offspring were PCR-screened for detection of cas9-generated mutations. Among the 50 screened adults, 47 had one visible PCR product of the expected size of the wild-type allele (Fig. 5C1), and three individuals had two PCR products [one with the expected size of the wild-type allele and a second smaller PCR product indicating a deletion (Fig. 5C2)]. DNA sequencing of the smaller band revealed a 52 bp deletion (Fig. 5D) at the sgRNA site in the *Ae-Forked* gene, resulting in a predicted frameshift, leading to a premature stop codon and a predicted truncated protein of 801 aa instead of the 1,342 aa in the wild-type protein (Fig. 5A, Forked knock-out). Our results showed that the truncated *Ae-Forked* mutant protein is a loss of function allele since it is missing two putative actin binding sites. Previously, it was shown that exon 3 (corresponding to amino acids 1,107 to 1,227 aa, Fig. 5B) and exon 5 (corresponding to amino acids 1,340 to 1,379) of the *Drosophila Forked* alternative splice form, were sufficient for *Forked* function in bristle development have independent actin-binding activity (black boxes in Fig. 5B)^20^. These actin-binding domains are located from amino acid 980 to 1,025 and from amino acid 1,127 to 1,229 aa in *Ae-Forked* protein (Fig. 5B, black boxes). Our truncated *Ae-Forked* mutant protein, which contains only 801 aa (see sgRNA location in Fig. 5B) instead of the 1,342 aa in the wild-type protein, lacks these domains. We mated G2 heterozygous mosquitoes and PCR-screened for homozygous *Ae-Forked* mutant individuals. We found that the *Ae-Forked* gene is essential for mosquito development, as all PCR-identified homozygous *Ae-Forked* mutants (Fig. 5C3) died as pharate adults (a pharate insect is one that has completed the metamorphosis from larva to adult, but is still within the pupa) that were partly emerged from their pupal case.

**Figure 6:**
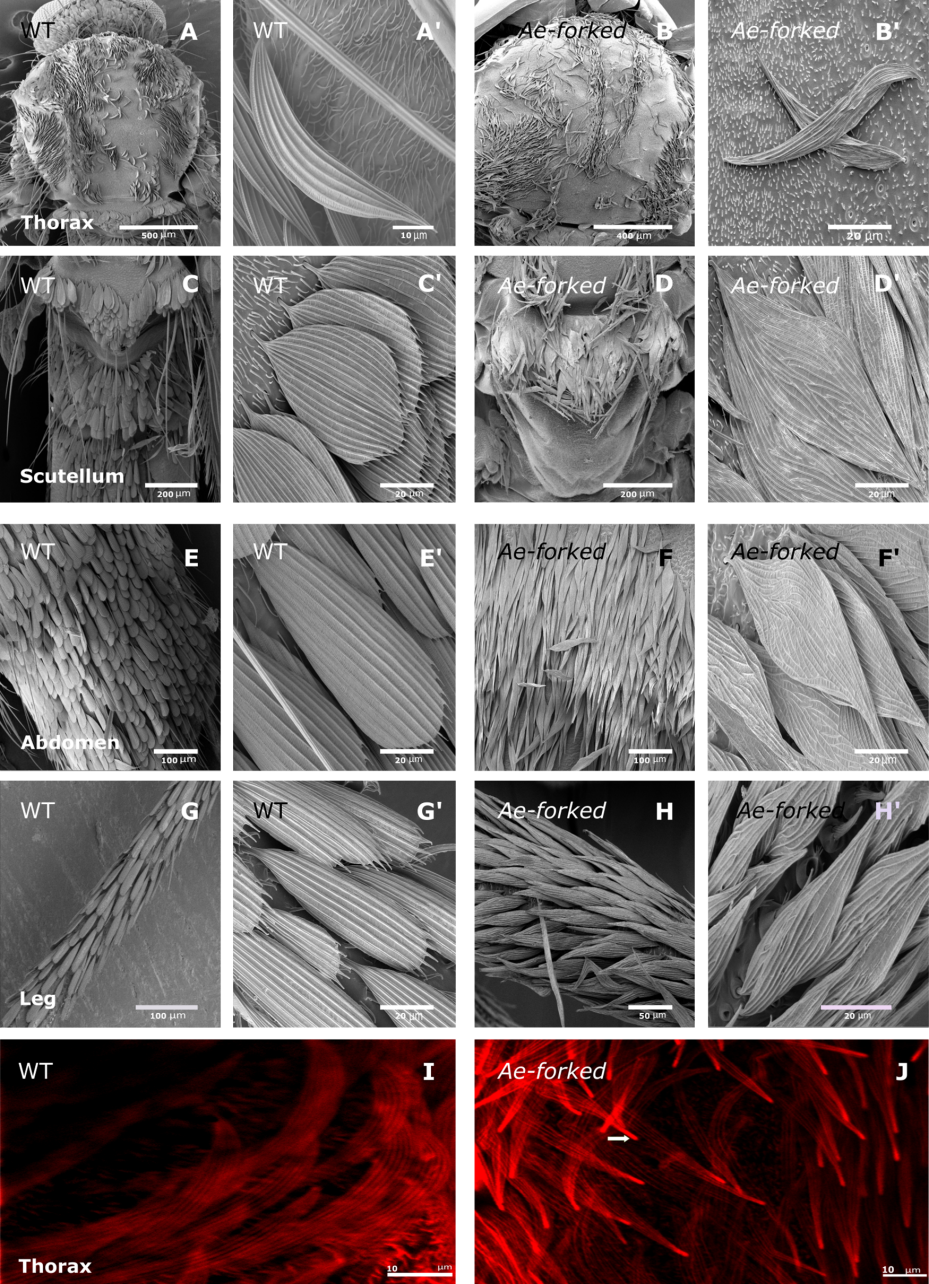
Scanning electron microscopy (SEM) and confocal microscopy images of scales from different tissues of WT and *Ae-Forked* pharate mutant mosquitoes, *Aedes aegypti*. Thorax of WT (**A**) and *Ae-Forked* pharate (**B**) mutant mosquitoes. Scutellum of WT (**C**) and *Ae-Forked* pharate mutant (**D**) mosquitoes. Abdomen of WT (**E**) and *Ae-Forked* pharate (**F**) mutant mosquitoes. Legs of WT (**G**) and *Ae-Forked* pharate (**H**) mutant mosquitoes. (**A**′,**C**′,**E**′,**G**′) high-magnification image of WT scales reveals that along each scale, there are longitudinal ridges which spread from the base of the scale to the tip. High-magnification image of scales from all tissues from *Ae-Forked* pharate mutants (**B**′,**D**′,**F**′,**H**′) revealed that the ridges on the scales are misoriented. In all spatulate scales (**D**′,**F**′,**H**′), the upper part was no longer broad, but instead tapered towards the tip, resembling the bristle tip. Scale bars are found on each image. Phalloidin staining (actin-red) of scales on the thorax from (**I**) WT and (**J**) *Ae-Forked* pupae 15 h APF. In *Ae-Forked* pupae, actin (arrows) accumulate at the tip of the scale.

Next, we used SEM to examine the nature of the defects in both the hairs, bristles, and scales in *Ae-forked* pharate mutant lines. Examination by SEM (Fig. 6) showed that scales were affected on the thorax (Fig. 6A,B) scutellum (Fig. 6C,D) abdomen (Fig. 6E,F), and leg (Fig. 6G,H). In mutant mosquito body parts that had scales with a spatulate shape such as the scutellum (Fig. 6C), abdomen (Fig. 6E), and leg (Fig. 6G), the upper part was no longer broad as in the wild type, but instead tapered towards the tip (Fig. 6D′,F′,H′). Examination of scales in all body parts revealed that, compared to the wild type which had longitudinal ridges (Fig. 6A′,C′,E′,G′), in *Ae-Forked* pharate mutants, all of the ridges were misoriented (Fig. 6B′,D′,F′,H′). To quantify the morphological changes in scale morphology in *Ae-Forked* pharate mutants, we focused our analysis on the scutellum. We found that compared to wild-type scales, the upper part of the scales (23.718 ± 0.960 µm) was significantly (t = − 20.799, df = 6, p << 0.0001) narrower in *Ae-Forked* pharate mutants (2.87 ± 0.287 µm, Table 2). On the other hand, in these mutants, the scales were significantly longer than in the wild type (78.75 ± 4.278 µm and 56.789 ± 3.891 µm, respectively, Table 2). Next, we analyzed scale actin-bundle organization during development by confocal microscope analysis following phalloidin staining. Closer examination of scales from the thorax of *Ae-forked* pharate mutants revealed that they were highly significantly narrower (3.3 ± 0.6, n = 21 scales from four pupae (Fig. 6J), as compared to both untreated pupae (7.6 ± 1 µm, Fig. 6I) and CD-treated pupae (5.4 ± 0.7, Fig. 4L). Moreover, a high accumulation of actin was also evident in the upper part scales from the thorax of *Ae-forked* pharate mutants (Fig. 6J, arrow).

**Table 2:** Bristle and scale measurement in WT and *Ae-Forked* pharate mutants.

Genotype	WT	*Ae-Forked* pharate mutants
No. of pupa	4	13
No. of bristles	15	31
Bristle length	255.082 ± 12.106*	165.299 ± 6.679
No. of pupa	6	6
No. of scales	21	30
Scale width—middle	25.385 ± 1.204	23.338 ± 1.617
Scale width—top	23.718 ± 0.960*	2.87 ± 0.287
Scale length	56.789 ± 3.891*	78.75 ± 4.278

*Represents a significant difference (p < 0.05) between genotypes (for a detailed description of statistical analyses performed, see “Materials and methods”).

**Figure 7:**
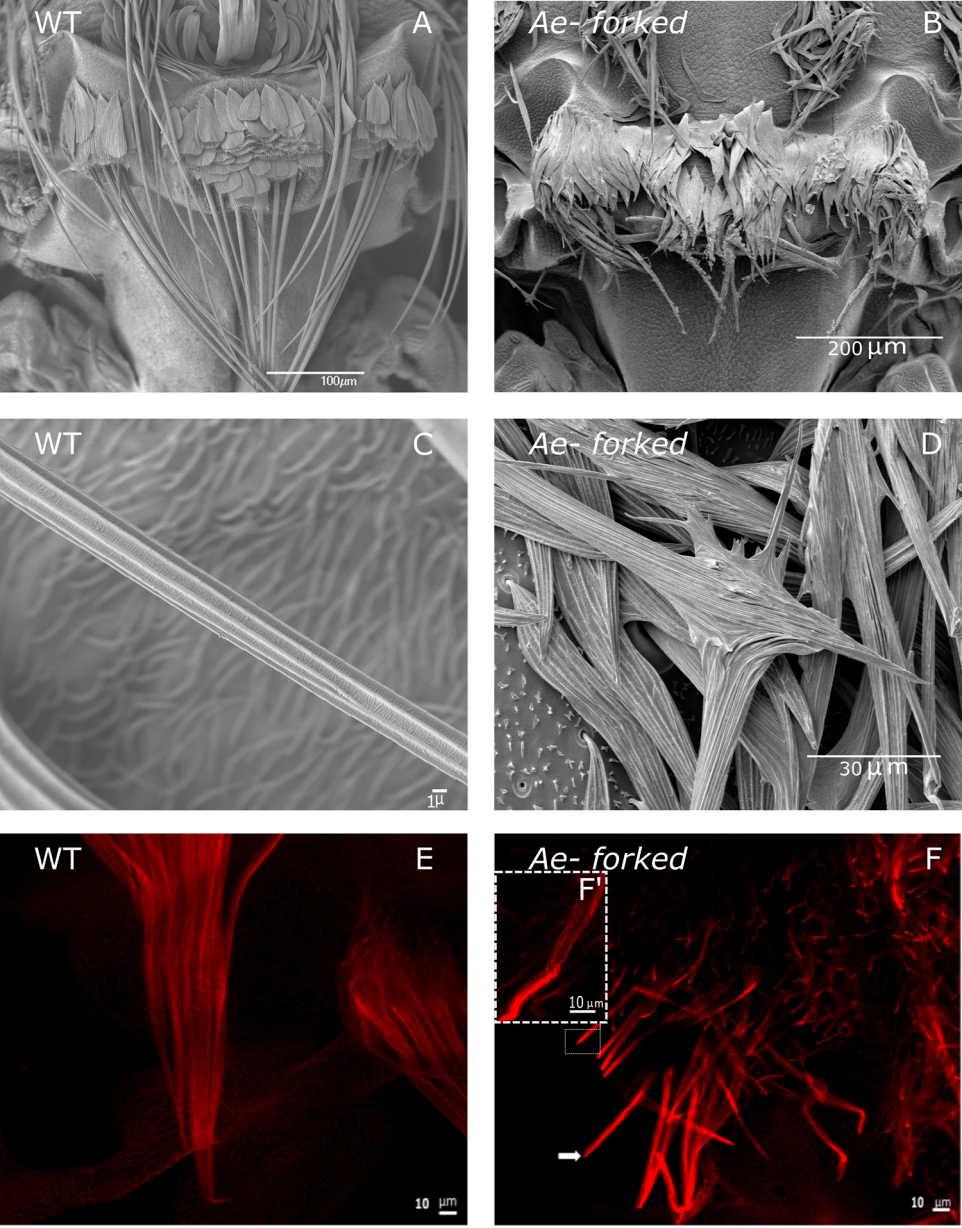
Scanning electron microscopy (SEM) and confocal microscopy images of bristles from different tissues of WT and *Ae-Forked* pharate mutant mosquitoes, *Aedes aegypti*. (**A**) WT scutellum, (**B**) *Ae-Forked* scutellum (**D**) In *Ae-Forked* pharate mutants, bristles are much shorter than in WT. (**C**,**D**) SEM image of WT (**C**) and *Ae-Forked* pharate mutant bristles (**D**). In WT, there are longitudinal ridges from the base of the bristle to the tip. In *Ae-Forked* pharate mutants, the bristle is split, and the ridges are misorienated. Scale bars are found on each image. (**E**,**F**) Phalloidin staining (actin-red) of bristles on the scutellum from (**E**) WT and *Ae-Forked* pupae (**F**,**F**′) 15 h APF. (**F**) Arrow points to actin accumulation on the tip of the bristle from *Ae-Forked* pharate mutants.

Since in *Drosophila*, the *Forked* gene affects both bristle and hair development, we tested whether these structures were affected in *Ae-Forked* pharate mutants. Analyzing bristle morphology in these mutants (Fig. 7B) revealed that bristles were significantly shorter (t = − 6.494, df = 5, p = 0.0013) in them (165.299 ± 6.679) compared to wild-type bristles (Fig. 7A; 255.082 ± 12.106 µm, Table 2). Closer examination revealed that these bristles lost their shape and also their regular ridge and valley pattern (Fig. 7C vs. Fig. 7D). To better understand the role of *Ae-Forked* on bristle actin-bundle organization, we used confocal microscope analysis following phalloidin staining. In contrast to wild type bristles (Fig. 7C), in *Ae-Forked* pharate mutants, phalloidin staining revealed a strong accumulation of actin on the upper part of the bristle (Fig. 7F, arrow), and similarly to CD-treated pupae, actin bundles were misoriented (Fig. 7F′).

**Figure 8:**
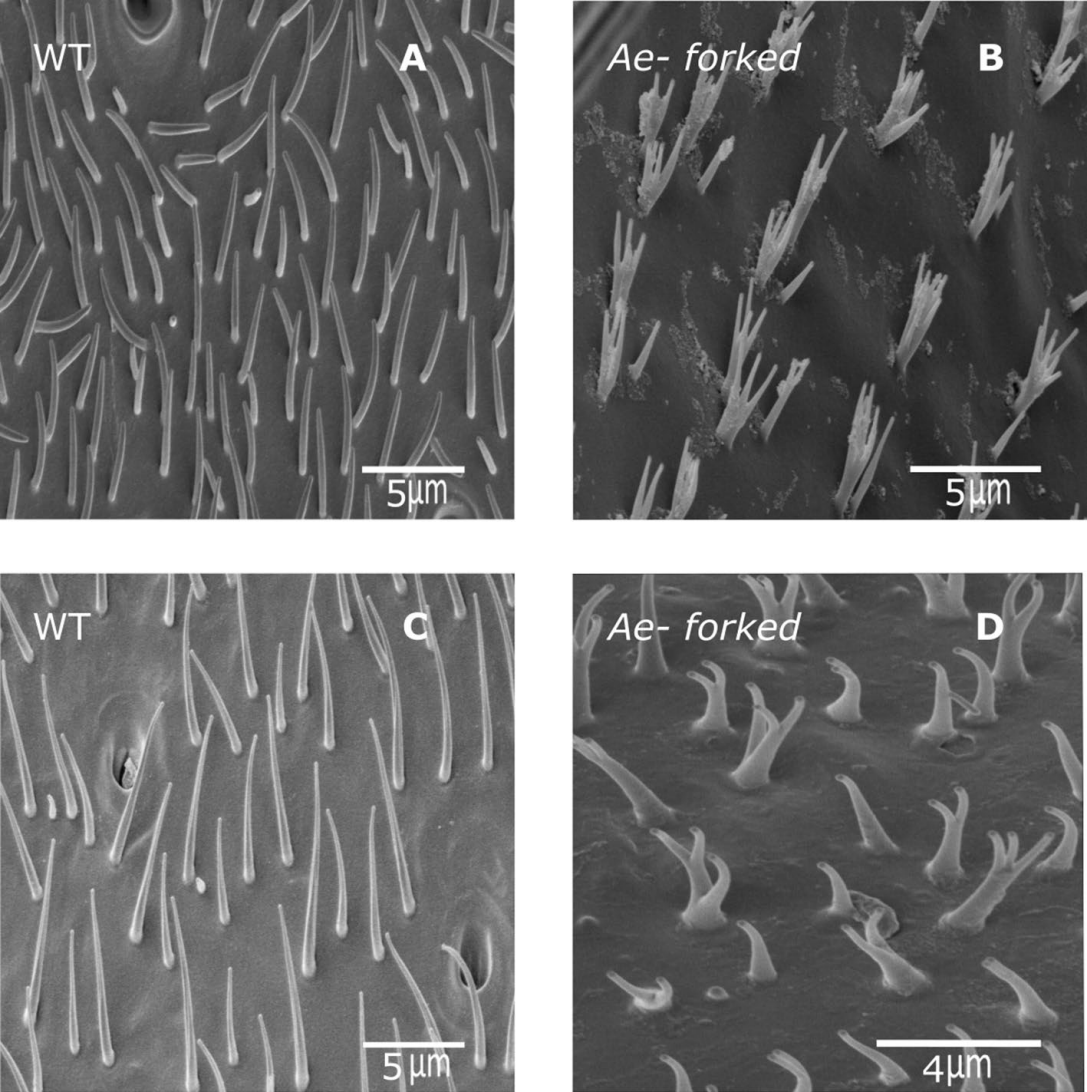
Scanning electron microscopy (SEM) images of hairs from different tissues of WT and *Ae-Forked* pharate mutant mosquitoes, *Aedes aegypti*. Hairs from the thorax (**A**,**B**) and scutellum (**C**,**D**) from mosquito tissues. WT (**A**,**C**) and *Ae-Forked* pharate mutants (**B**,**D**) mosquitoes. In both WT tissues, hairs are cylindrical in shape which tapers towards the tip. In *Ae-Forked* pharate mutants, hairs are shorter and split. Scale bars are found on each image.

Next, we analyzed hair structure on the thorax (Fig. 8A,B) and scutellum (Fig. 8C,D) and found that hair morphology in *Ae-Forked* pharate mutants was affected with splintered ends compared to the tapered cylinder morphology of the wild type; (Fig. 8A,C vs. Fig. 8B,D). Similar to bristles, the length of hairs were significantly shorter in *Ae-Forked* pharate mutants both on the thorax (wild-type—4.289 ± 0.433 µm, mutant—2.135 ± 0.127 µm, t = − 4.776, df = 2, p = 0.041) and on the scutellum (wild-type—5.836 ± 0.102 µm, 2.474 ± 0.183 µm, t = − 16.061, df = 6, p < 0.0001).

## Discussion

### Mosquito scales undergo drastic morphology and actin cytoskeleton changes during development

In this study, we structurally characterized *Aedes aegypti* scale development. We showed that at early developmental stages, scales (like bristles) are cylindrical in shape, with membrane-associated actin bundles. Thus, bristles and scales share similar cytoskeleton organization. However, during late scale development, scales undergo drastic morphological changes, which appear in two stages. First, the cylindrical shape changes to a flatter shape, and second, longer membrane invagination (presumably the future scale ridges) appears on one side of the scale. In bristles, on the other hand, one obvious morphological change occurs—the appearance of ridges and valleys. This change corresponds to the appearance of longer membrane invagination in scales. In both cases, the ridges or longer membrane invaginations correspond to sites on the cell membrane where actin bundles are absent.

The organization of actin bundles also changes during development. Three main phases in actin-bundle formation can be described. At early stages, almost the entire circumference of the scale is covered with approximately 20 actin bundles. In the second phase, the actin bundles grow in size and become triangular in shape as bristle actin bundles. In the third phase, the actin bundles are organized asymmetrically, where one side contains large rounded actin bundles, and on the opposite side, the actin bundles are smaller and flatter. The same kind of asymmetrical actin-bundle organization can also be found in bristles, where they are large in a cross-sectional area on the inferior surface and small or, in some cases, absent on the superior surface^21^. Whereas the first and second phase of actin-bundle organization is found in the cylinder shape of the scale, the third phase is associated with the flat shape of the scale with longer membrane invagination. Thus, although actin-bundle organization between scales and bristles showed some similarity, we believe that the different organization of the actin cytoskeleton between these two homologous structures dictates their different morphology.

### Temporal and spatial scale organization

In this study, we revealed that the timing of scale initiation between different body parts (scutellum and legs) is different. On the legs, scale buds occur early on at 5 h APF compared to scales on the scutellum, which initiate at 7 h APF. For our experimental purposes, it was important to reveal the exact timing of bud initiation, so we could interfere with scale growth early on. Similar phenomena have been observed on butterfly wings, where it was shown that longer scales will bud earlier and elongate more quickly than shorter scales^11^. In parallel to their different bud initiation time, we also noticed that the pattern of scale development between these two body parts is different. On the legs, but not on the scutellum, we noticed that scales occur in clusters of four cells that differed in size. This unique scale organization on the leg raises an intriguing question regarding the molecular mechanism underlying the differential size between the scales in each cluster. To address this, we examined earlier stages, and found that all different-sized scales within the cluster grew simultaneously. Still our preliminary data were not sufficient to conclude whether all four cells within the cluster have different or similar identity. To answer this, further experiments are needed. Still, our results reveal that the pattern of scale development is different between different mosquito body parts, suggesting that there are multiple mechanisms that control mosquito scale development.

### In contrast to mosquito bristles and hairs and butterfly scales, actin bundles in mosquitoes play a different role in scale elongation

One of the main requirements of actin bundles in *Drosophila* bristles and hairs and in butterfly scales is elongating cell extension. In *Drosophila*, treatment with CD before and after bristle initiation affects bristle elongation^22,23^. In the butterfly *Vanessa cardui*, inhibition of actin-bundle formation before and after bud initiation reveals that actin bundles are required for initial scale elongation^11^. In this study, we inhibited actin-bundle formation before scale and bristle initiation and found that this treatment affected scale and bristle morphology. As expected, the regular ridge pattern of both cell types was strongly affected. But, whereas bristles were significantly shorter than in controls, scales were significantly narrower and longer than control scales.

These results led us to further investigate the role of actin bundles in shaping the mosquito's epidermal cellular extension by mutating one of the known actin-bundle proteins, *Forked*. *Drosophila Forked* is required for actin-bundle formation both for bristles^6^ and hairs^24^. In *Drosophila Forked* mutants, bristles are shorter (50% as the long as the wild type), thicker and twisted, and in some cases, they exhibit forked tips^21,25^. We knocked out the mosquito *Forked* homologue, *Ae-Forked*, and found that, similar to *Drosophila* bristles, the bristles were significantly shorter than the wild type with misorganized longitudinal ridges. On the other hand, in *Ae-Forked* pharate mutants, scales were significantly longer than in the wild type, and they had lost their ridge pattern. The most affected region in the scales was the tip. In all three body parts examined, the broad distal region became tapered at the bristle tip. In total, our results (both inhibition of actin polymerization and also from *Ae-Forked* pharate mutants) demonstrate that actin bundles play differential roles in scales compared to bristles. In bristles, actin bundles are required for cell elongation, and in scales, actin bundles are required for width formation.

As described above, the scale morphology defects in CD-treated pupae and in *Ae-Forked* pharate mutants affect scale widening with a more prominent effect in the upper part of the scales, but it is not clear why. What could be the reason for this? Widening of the scale occurs first during the cylindrical to flatter shape, and then slowly during scale development (from 12 h APF and later).What happens to scale actin-bundle organizations during development? At an early stage, before scales flatten, almost the entire circumference of the scale is covered with actin bundles. After scale cells flatten, the actin bundles are organized asymmetrically, where one side contains large actin bundles, and on the opposite side, the actin bundles are smaller. Our confocal analysis on actin organization during scale development revealed that both in CD-treated pupae and in *Ae-Forked* pharate mutants, actin accumulates abnormally at the scale tip. The question remains: At what stage of cell widening are actin bundles required? Is it in the first stage, where the cylindrical shape becomes flatter or at a later stages of development? Given that the scale tip is most affected by disturbed actin organization, we postulate that affecting actin-bundle organization by inhibiting actin polymerization chemically or by *Ae-Forked* gene deletion before the scale is flattened, results in narrower scales. Thus, the unique membrane-associated actin-bundle organization in scales contributes to widening of the scale.

## Supplementary information

Supplementary Information.

## Data Availability

The datasets analyzed during the current study are available from the corresponding authors upon reasonable request.
