# Supplementary material for: Actin bundles play a different role in shaping scales compared to bristles in the mosquito Aedes aegypti
Source: Sci Rep. 2020 Sep 10;10:14885. doi: 10.1038/s41598-020-71911-0 (PMC7483531; doi:10.1038/s41598-020-71911-0)
Supplement: Supplementary file 1 — Supplementary Information. [file 41598_2020_71911_MOESM1_ESM.docx]

Actin bundles play a different role in shaping scales compared to bristles in the mosquito *Aedes aegypti*

Sanja Djokic^1^, Anna Bakhrat^1^, Ido Tsurim^2^, Nadya Urakova^3^, Jason L. Rasgon^3, 4, 5^, and Uri Abdu^1,*.^

^1^Department of Life Sciences, Ben-Gurion University of the Negev, Beer Sheva 84105, Israel

^2^Department of Life Sciences, Achva Academic College, Israel

^3^Department of Entomology, The Pennsylvania State University, University Park, PA, United States of America

^4^The Huck Institutes of the Life Sciences, The Pennsylvania State University, University Park, PA, United States of America

^5^Center for Infectious Disease Dynamics, The Pennsylvania State University, University Park, PA, United States of America

**^*^**Corresponding author: abdu@bgu.ac.il


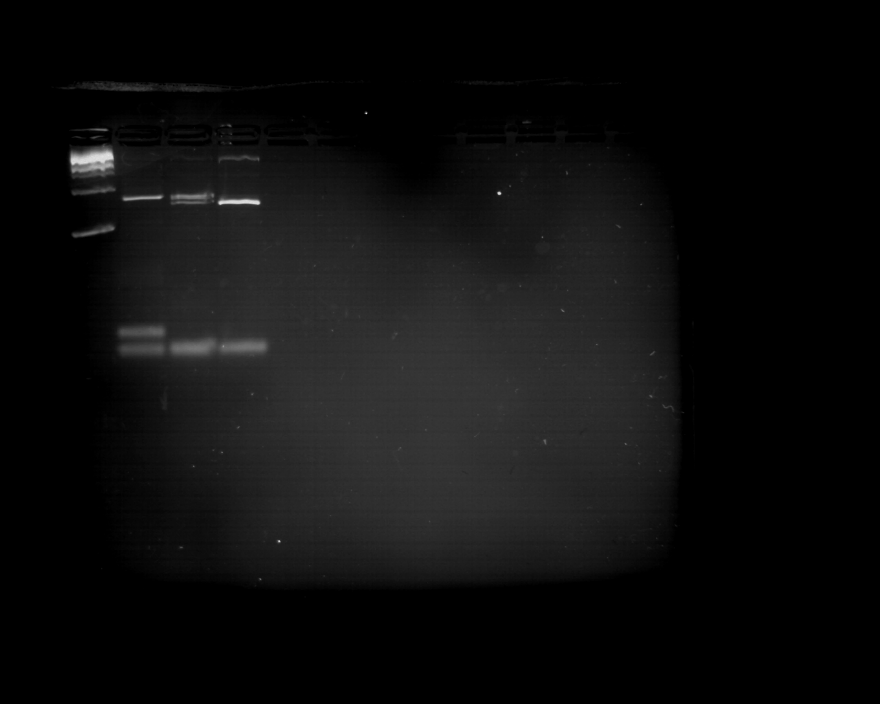
**entire figure related to Figure 5B.**

PCR analysis of WT and mutant mosquito lines. 1) PCR on WT mosquitos

reveal PCR product of in size. 2) PCR on G1 putative mutant heterozygous

lines reveal 2 PCR products, one in the same size as in WT the second one

is smaller in size. 3) PCR on homozygous G2 Ae-forked line showing only

one band which represent the deletion of 52 bp.
